# Fixação combinada intramedular-externa com fios de Kirschner para fraturas das falanges: Descrição de técnica cirúrgica

**DOI:** 10.1055/s-0046-1824572

**Published:** 2026-10-06

**Authors:** Gabriel Ifran Alves, Antônio Lourenço Severo, Marcelo Barretos de Lemos, Osvandré Lech, Felipe Kowaleski, Ivânio Tagliari

**Affiliations:** 1 Instituto de Ortopedia e Traumatologia (IOT), Hospital São Vicente de Paulo (HSVP), Passo Fundo, RS, Brasil

**Keywords:** falanges dos dedos da mão, fios de Kirschner, fixação intramedular de fraturas, fixadores externos, fraturas ósseas, external fixators, finger phalanges, fracture fixation, intramedullary, fractures, bone, Kirschner wires

## Abstract

A mobilização precoce é fundamental para a recuperação funcional após fraturas das falanges, mas muitos métodos de fixação ainda exigem imobilização prolongada, o que aumenta o risco de rigidez articular. Descrevemos uma técnica simples, segura e de baixo custo, que combina fios de Kirschner intramedulares e ligação externa, adaptada do método de Ulson, originalmente desenvolvido para fraturas do rádio distal. O procedimento consiste em redução fechada sob fluoroscopia, seguida da introdução de 2 fios intramedulares de 1,0 mm, cujas extremidades externas são dobradas e conectadas, e formam um quadro estável que permite mobilização imediata sem necessidade de imobilização. A técnica proporcionou fixação estável, consolidação óssea confiável e reabilitação precoce, com baixa taxa de complicações. Trata-se de uma opção prática e reprodutível, especialmente útil em contextos com recursos limitados.

## Abstract (en)

Although early mobilization is essential for functional recovery after phalangeal fractures, many fixation methods still require prolonged immobilization, increasing the risk of joint stiffness. We herein describe a simple, safe, and low-cost technique that combines intramedullary Kirschner wires (K-wires) with external connection, adapted from the Ulson method, which was originally developed for distal radius fractures. The procedure consists of closed reduction under fluoroscopy followed by the insertion of two 1.0-mm intramedullary wires. The external ends of these wires are bent and connected, forming a stable frame that enables immediate mobilization without the need for immobilization. The technique provided stable fixation, reliable bone healing, and early rehabilitation, with a low complication rate. It represents a practical and reproducible option, especially useful in resource-limited settings.

## Introdução

As fraturas das falanges da mão são lesões frequentes e exigem manejo preciso para restaurar a função e evitar rigidez. Fraturas estáveis geralmente evoluem bem com tratamento conservador, ao passo que fraturas instáveis ou desviadas requerem estabilização cirúrgica. Métodos tradicionais, como fios de Kirschner, parafusos e miniplacas, apresentam limitações relacionadas à imobilização prolongada, maior dissecção de tecidos moles ou custos elevados em ambientes com recursos limitados. 1 2 Para superar essas limitações, descrevemos uma técnica simples e acessível que combina fixações intramedular e externa com fios de Kirschner, o que proporciona alinhamento estável e permite mobilização precoce em fraturas das falanges proximal e média. 3 4

## Técnica cirúrgica

A técnica é indicada para fraturas diafisárias ou metafisárias extra-articulares das falanges proximal e média que necessitam de estabilização cirúrgica, nas quais se deseja mínima dissecção de partes moles e mobilização precoce. 5 6

O procedimento pode ser realizado sob anestesia regional ou geral, com o paciente em posição supina e o membro superior apoiado sobre mesa auxiliar. A redução fechada é obtida sob controle fluoroscópico, aplicando-se tração suave e manipulação digital. Dois fios de Kirschner de 1,0 mm são introduzidos de forma intramedular a partir dos pontos de entrada medial e lateral na base da falange, atravessando a linha de fratura e mantendo alinhamento paralelo. Dependendo do traço, os fios também podem ser inseridos de forma retrógrada. As extremidades externas são dobradas para formar uma estrutura retangular e conectadas com fita microporosa, o que fornece estabilidade adicional. A redução e a fixação são confirmadas por fluoroscopia, e aplica-se somente curativo simples, sem imobilização ( Figs. 1 2 3 4 ). Essa montagem baseia-se no método de Ulson, originalmente descrito para fraturas do rádio distal, aqui adaptado para fraturas dos dedos da mão. Na técnica apresentada, os fios de Kirschner podem ser inseridos em configuração paralela ou cruzando-se no interior da diáfise, dependendo do padrão da fratura e do tamanho do osso. Em ambas as configurações, o princípio biomecânico permanece o mesmo, e funciona como um sistema elástico que gera compressão centrípeta no foco da fratura. Curativos simples são mantidos para conforto no pós-operatório.

**Fig. 1:**
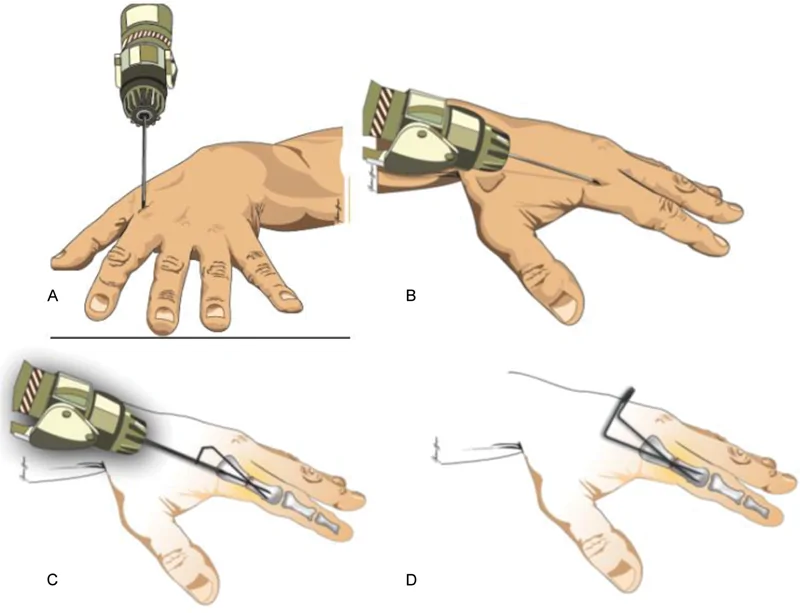
( **A** ) Vista frontal da introdução do fio metálico; ( **B** ) vista lateral; ( **C** ) redução e fixação da fratura; ( **D** ) aspecto final tipo “goleira”, estabilizado de forma a permitir mobilização ativa precoce.

**Fig. 2:**
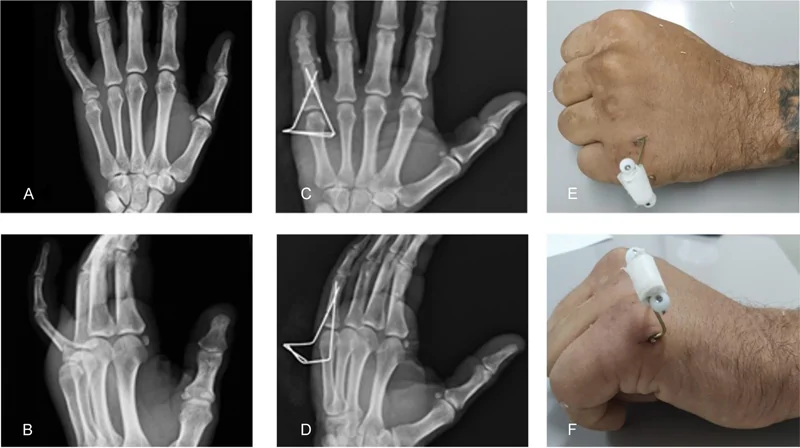
Caso ilustrativo. ( **A,B** ) Fratura da base da falange proximal do quinto dedo com desvio dorsal; ( **C,D** ) radiografias no pós-operatório imediato; ( **E,F** ) aspecto externo, demonstrando a capacidade do paciente de realizar mobilização. Neste caso, foi utilizado temporariamente um pequeno protetor esférico plástico nas extremidades externas dos fios de Kirschner para proteção da pele. Esse componente não contribui para a estabilidade da montagem e não é utilizado rotineiramente.

**Fig. 3:**
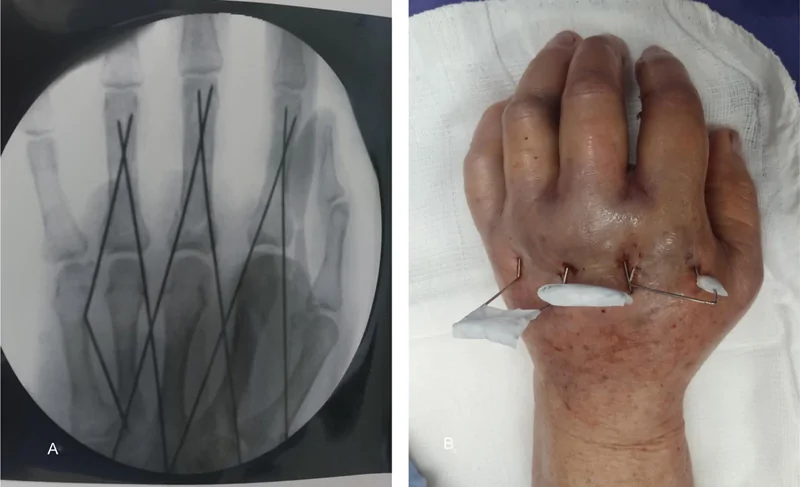
( **A,B** ) Paciente com fraturas das falanges proximais do segundo ao quarto dedos tratados pelo método combinado de fixações interna e externa, ilustrando a configuração do implante no pós-operatório imediato. As radiografias pré-redução e de controle final deste caso não estavam disponíveis.

**Fig. 4:**
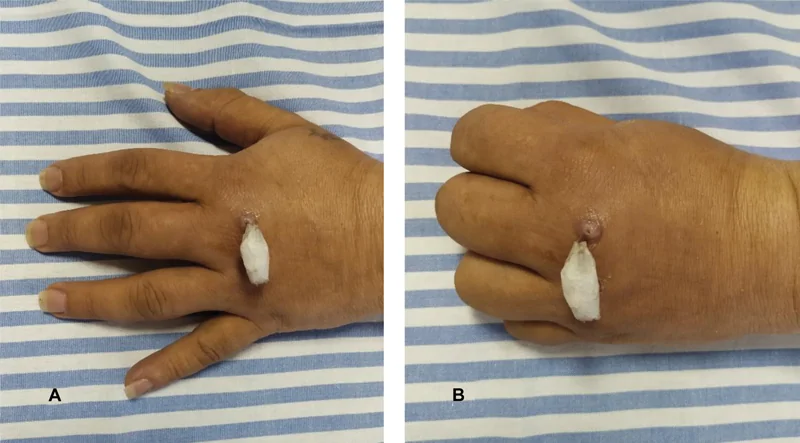
( **A** ) Dedos em extensão e ( **B** ) em flexão, demonstrando clinicamente a mobilidade ativa permitida em um paciente com fratura da falange proximal do dedo médio tratado pelo método descrito aqui. As imagens têm caráter ilustrativo, e somente visam demonstrar a capacidade de mobilização ativa proporcionada pela técnica.

A mobilização ativa e passiva dos dedos é iniciada imediatamente após a cirurgia. Radiografias seriadas são obtidas a cada 2 semanas para acompanhamento da consolidação. Os fios de Kirschner são removidos assim que a consolidação óssea é confirmada radiograficamente, geralmente entre a quinta e a sexta semanas pós-operatórias. Após a retirada dos fios, o paciente continua os exercícios de amplitude de movimento até a recuperação completa.

## Desfecho

A técnica descrita foi aplicada em uma série consecutiva de 18 pacientes com fraturas das falanges proximal e média, mais frequentemente envolvendo os dedos mínimo e anular. Quinze pacientes completaram o seguimento clínico. Todas as fraturas consolidaram entre 5 e 6 semanas, com recuperação precoce da mobilidade e baixa taxa de complicações. Três casos de infecção superficial no trajeto dos pinos foram tratados de forma conservadora, sem perda de redução, necessidade de remoção da síntese ou atraso de consolidação. 7

A fixação combinada intramedular-externa oferece maior estabilidade rotacional, o que preserva a simplicidade e o caráter minimamente invasivo da fixação percutânea. A conexão externa dos fios permite mobilização precoce sem necessidade de imobilização, o que favorece menor rigidez articular e menor risco de aderências tendíneas. Além disso, o método apresenta baixo custo e fácil reprodutibilidade, características que beneficiam sua aplicação rotineira em diferentes cenários assistenciais. 8 9 Um aspecto específico dessa configuração é a geração de forças centrípetas que promovem compressão na linha de fratura. 10 Neste estudo, a técnica foi aplicada a fraturas diafisárias e metafisárias extra-articulares das falanges.

Apesar dos bons resultados, é essencial o controle rigoroso por fluoroscopia e o posicionamento preciso dos fios para evitar penetração articular. Com o acúmulo de experiência clínica, será possível definir melhor as indicações e os resultados em longo prazo dessa técnica.

## Conclusão

A fixação combinada interna-externa com fios de Kirschner representa uma opção segura, reprodutível e de baixo custo para o tratamento de fraturas instáveis das falanges proximal e média. Permite mobilização ativa imediata e consolidação óssea confiável, com baixa taxa de complicações. O método foi conceitualmente adaptado da técnica de Ulson, originalmente desenvolvida para fraturas do rádio distal – uma importante contribuição brasileira à cirurgia ortopédica –, e sua aplicação bem-sucedida em fraturas das falanges reforça o potencial de adaptação de técnicas consolidadas a novos contextos anatômicos e clínicos.
